# High-intensity interval training for 12 weeks improves cardiovascular autonomic function but not somatosensory nerve function and structure in overweight men with type 2 diabetes

**DOI:** 10.1007/s00125-022-05674-w

**Published:** 2022-03-11

**Authors:** Gidon J. Bönhof, Alexander Strom, Maria Apostolopoulou, Yanislava Karusheva, Theresia Sarabhai, Dominik Pesta, Michael Roden, Dan Ziegler

**Affiliations:** 1 Institute for Clinical Diabetology, German Diabetes Center, Leibniz Center for Diabetes Research at Heinrich Heine University Düsseldorf, Düsseldorf, Germany; 2 Department of Endocrinology and Diabetology, Medical Faculty and University Hospital Düsseldorf, Heinrich Heine University Düsseldorf, Düsseldorf, Germany; 3 German Center for Diabetes Research (DZD), Partner Düsseldorf, München Neuherberg, Germany

**Keywords:** Autonomic neuropathy, Cardiac autonomic function, Diabetic neuropathy, Exercise training, High-intensity interval training, HIIT

## Abstract

**Aims/hypothesis:**

It remains unclear whether and which modality of exercise training as a component of lifestyle intervention may exert favourable effects on somatosensory and autonomic nerve tests in people with type 2 diabetes.

**Methods:**

Cardiovascular autonomic and somatosensory nerve function as well as intraepidermal nerve fibre density (IENFD) were assessed in overweight men with type 2 diabetes (type 2 diabetes, *n* = 20) and male glucose-tolerant individuals (normal glucose tolerance [NGT], *n* = 23), comparable in age and BMI and serving as a control group, before and after a supervised high-intensity interval training (HIIT) intervention programme over 12 weeks. Study endpoints included clinical scores, nerve conduction studies, quantitative sensory testing, IENFD, heart rate variability, postural change in systolic blood pressure and spontaneous baroreflex sensitivity (BRS).

**Results:**

After 12 weeks of HIIT, resting heart rate decreased in both groups ([mean ± SD] baseline/12 weeks: NGT: 65.1 ± 8.2/60.2 ± 9.0 beats per min; type 2 diabetes: 68.8 ± 10.1/63.4 ± 7.8 beats per min), while three BRS indices increased (sequence analysis BRS: 8.82 ± 4.89/14.6 ± 11.7 ms^2^/mmHg; positive sequences BRS: 7.19 ± 5.43/15.4 ± 15.9 ms^2^/mmHg; negative sequences BRS: 12.8 ± 5.4/14.6 ± 8.7 ms^2^/mmHg) and postural change in systolic blood pressure decreased (−13.9 ± 11.6/−9.35 ± 9.76 mmHg) in participants with type 2 diabetes, and two heart rate variability indices increased in the NGT group (standard deviation of R–R intervals: 36.1 ± 11.8/55.3 ± 41.3 ms; coefficient of R–R interval variation: 3.84 ± 1.21/5.17 ± 3.28) (all *p*<0.05). In contrast, BMI, clinical scores, nerve conduction studies, quantitative sensory testing, IENFD and the prevalence rates of diabetic sensorimotor polyneuropathy and cardiovascular autonomic neuropathy remained unchanged in both groups. In the entire cohort, correlations between the changes in two BRS indices and changes in $\dot{V}{\mathrm{O}}_{2\max }$ over 12 weeks of HIIT (e.g. sequence analysis BRS: *r* = 0.528, *p*=0.017) were observed.

**Conclusions/interpretation:**

In male overweight individuals with type 2 diabetes, BRS, resting heart rate and orthostatic blood pressure regulation improved in the absence of weight loss after 12 weeks of supervised HIIT. Since no favourable effects on somatic nerve function and structure were observed, cardiovascular autonomic function appears to be more amenable to this short-term intervention, possibly due to improved cardiorespiratory fitness.

**Graphical abstract:**

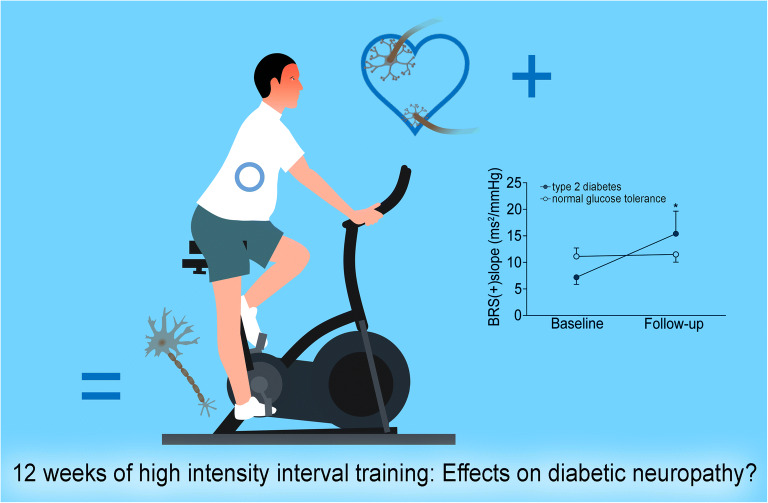

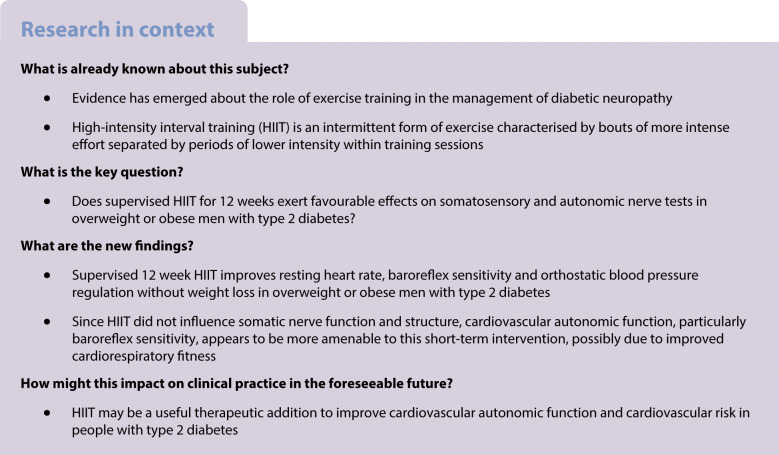



## Introduction

Diabetic sensorimotor polyneuropathy (DSPN) and cardiovascular autonomic neuropathy (CAN) are substantial contributors to an increased morbidity and mortality risk and reduced quality of life in people with diabetes [1]. Approximately 30% of individuals with diabetes are affected by DSPN [2]. Peripheral nerve damage due to DSPN results mainly in distal sensorimotor deficits and/or neuropathic symptoms such as paraesthesia, dysesthesias, numbness, muscle weakness and neuropathic pain. The prevalence of CAN in individuals with diabetes has been estimated to vary between 16% and 31% depending on the diagnostic criteria used [2]. Early CAN is characterised by reduced, predominantly vagally mediated, heart rate variability (HRV), increasing the risk of clinical features such as resting tachycardia, orthostatic hypotension, exercise intolerance and silent myocardial ischaemia [3]. Both DSPN and CAN may develop insidiously with a considerable heterogeneity of clinical features and complications, while the complex multifactorial pathogenesis remains incompletely understood [3].

Despite its major clinical impact, diabetic neuropathy remains a frequently underdiagnosed and undertreated condition [4, 5]. Current options to treat peripheral or autonomic manifestations of diabetic neuropathy are limited and primarily based on symptomatic management [1]. However, symptomatic pharmacotherapy recommended for painful DSPN is often insufficient to alleviate neuropathic pain and may lead to significant side effects. While long-term near-normoglycaemia has been shown to at least partially prevent the development of or delay the progression of DSPN or CAN in type 1 diabetes, this goal is still not achievable in many patients, and there is no clear evidence for such a favourable effect of intensive diabetes therapy in people with type 2 diabetes [2].

In recent years, evidence has emerged about the role of exercise training (ET) in the management of DSPN and CAN [6]. Several studies examined the effects of different forms of ET, focusing on strength, endurance or balance, on DSPN measures such as NCS, small fibre density, sensorimotor function and neuropathic symptoms [7]. Notably, a few clinical trials reported improved NCS in diabetic individuals with or without diabetic neuropathy [8–10]. Others indicated that neuropathic symptoms including paraesthesia, neuropathic pain and balance were reduced after exercise [11, 12]. More studies are available, mostly on balance ET, that reported improved performance in balance tests [12–16]. However, the current evidence is not sufficient to draw firm conclusions about the effectiveness of different ET strategies to improve measures of peripheral nerve function or even reverse DSPN.

It is known that regular aerobic ET may beneficially affect cardiovascular measures including sympathovagal balance, resting heart rate and blood pressure in healthy individuals [17]. Evidence from clinical trials suggests that ET programmes may improve HRV in patients with type 2 diabetes. While the frequency of exercise units seems to be an important factor, short intervention programmes over several months only may be less successful in improving HRV [18]. The baroreflex function assessed by spontaneous baroreflex sensitivity (BRS) modulates cardiac contractility, heart rate and peripheral vascular resistance in response to arterial blood pressure fluctuations [19]. It represents a prognostic index of CVDs and may be a promising marker of CAN in patients with diabetes [19]. Beneficial effects of physical exercise on circulation may be attributed in part to improved baroreflex function [20].

High-intensity interval training (HIIT) is an intermittent form of exercise characterised by bouts of more intense effort separated by periods of lower intensity within a single training session [21]. Evidence has accumulated that HIIT may exert beneficial effects on body composition and cardiorespiratory fitness with a lower time commitment and exercise volume compared with moderate-intensity training [22, 23]. However, no study has hitherto assessed the effects of HIIT on cardiovascular autonomic and somatosensory nerve function and intraepidermal nerve fibre density (IENFD) in type 2 diabetes individuals in comparison with people with normal glucose tolerance (NGT). In the present study we hypothesised that HIIT may exert favourable effects on distinct peripheral nerve tests in overweight or obese men with and without type 2 diabetes.

## Methods

### Study participants

Twenty participants with type 2 diabetes and 23 with NGT from the Effect of High-intensity Low-volume Training on Insulin Sensitivity in Type 2 Diabetes (HIT) study cohort (ClinicalTrial.gov registration no. NCT02039934) were included in the present study [24]. The study was approved by the local ethics committee of Heinrich Heine University, Düsseldorf, Germany and was conducted in accordance with the Declaration of Helsinki. Written informed consent was obtained from all participants prior to participation. Inclusion criteria were: age between 30 and 65 years, sedentary lifestyle and BMI ≥25 kg/m^2^. Type 2 diabetes was defined according to ADA criteria [25], while individuals with NGT underwent a standardised 75 g OGTT to exclude impaired glucose tolerance, impaired fasting glucose and diabetes.

Individuals with an acute infection within the last 2 weeks prior to the exercise intervention, autoimmune diseases and immune suppressive diseases, renal insufficiency, CVD, anaemia, disorders of wound healing or blood clotting, thyroid dysfunction and psychiatric disorders were excluded from the study. Further exclusion criteria were participation in another clinical study within the last 2 months before the investigation; medication with immunomodulating drugs, thiazolidinediones or insulin; current cigarette smoking; alcohol consumption >30 g/day; illegal drug abuse; night shift working; known hypersensitivity to local anaesthetics; history of cancer; lung diseases; performing systematic endurance training (>once per week >60 min); $\dot{V}{\mathrm{O}}_{2\max }$ <20 ml min^−1^ kg^−1^; orthopaedic disorders; and musculoskeletal diseases.

### HIIT

After the baseline assessment, all participants performed fully supervised HIIT on 3 non-consecutive days per week on a cycle ergometer for 12 weeks. Training sessions lasted 35 min including warm-up and cool-down periods and consisted of four intervals over 4 min at 90% of the individual’s maximum heart rate, determined during a baseline spiroergometry, alternating with 3 min intervals of recovery at 70% of the maximum heart rate. Participants were instructed not to alter other lifestyle habits and to maintain their body weight within ±5% of the initial body weight to exclude metabolic effects of weight reduction [24]. After 12 weeks, all baseline tests were repeated.

### Somatosensory nerve function

Peripheral nerve function was assessed as previously described [26, 27] at a skin temperature of 33–34°C using surface electrodes (Nicolet VikingQuest, Natus Medical, San Carlos, CA, USA). Motor nerve conduction velocity (MNCV) was measured in the median, ulnar and peroneal nerves. Sensory nerve conduction velocity (SNCV) was measured in the median, ulnar and sural sensory nerves. Sensory nerve action potential (SNAP) was determined in the sural nerve. Quantitative sensory testing (QST) included vibration perception thresholds (VPTs) at the metacarpal aspect of the hand and at the medial malleolus (Vibrameter, Somedic, Stockholm, Sweden) and thermal detection thresholds (TDTs) to warm and cold stimuli, which were determined at the dorsum of the foot (TSA-II NeuroSensory Analyzer, Medoc, Ramat Yishai, Israel). Clinical neuropathy scores included the Neuropathy Disability Score (NDS) and the Neuropathy Symptom Score (NSS) [28]. The diagnosis of DSPN was confirmed according to the Toronto Diabetic Neuropathy Expert Group consensus statement as the combination of an abnormal nerve conduction and/or reduced IENFD with signs and/or symptoms of neuropathy after exclusion of other causes [29].

### IENFD

Skin punch biopsy specimens (3 mm) were taken under local anaesthesia at the distal-lateral calf to determine IENFD as previously described [27].

### Cardiovascular autonomic nerve function

Cardiovascular autonomic function tests were performed to determine HRV indices during spontaneous breathing over 5 min (very low-frequency [VLF], low-frequency [LF] and high-frequency [HF] power; standard deviation of R–R intervals [SDNN]; coefficient of R–R interval variation [CVRR]), the lying to standing maximum/minimum 30:15 ratio (30:15 ratio) and the ratio in response to a Valsalva manoeuvre (Valsalva ratio) using a VariaCardio TF5 system (MIE, Leeds, UK) according to the recommendations of the Task Force of the European Society of Cardiology and the North American Society of Pacing and Electrophysiology [30] as previously described [19]. The systolic blood pressure response to standing up (postural change in systolic blood pressure [ΔSBP]) was measured over 3 min. Borderline CAN was assumed if two out of seven indices were abnormal, while definite CAN was diagnosed if at least three indices were abnormal, according to the recommendations of the CAN Subcommittee of the Toronto Consensus Panel on Diabetic Neuropathy [31].

### BRS

Continuous plethysmographic arterial measurements of spontaneous changes in systolic blood pressure and R–R intervals were recorded at the middle finger using a Finometer MIDI device (Finapres Medical Systems, Enschede, the Netherlands) to compute established indices of spontaneous BRS using commercially available software (Beatscope Easy version 2.1, Finapres Medical Systems, Enschede, the Netherlands; Nevrokard BRS Analysis version 6.3.0, Nevrokard, Izola, Slovenia) as previously described [19, 32]. Sequence analysis included positive-sequences BRS [BRS(+)slope], negative-sequences BRS [BRS(−)slope] and all-sequences BRS (BRS-allSeq), while BRS spectral analysis included the LF and HF bands and the mean of both bands. Indices of spectral analysis could not be computed in a significant proportion of participants (39.5%, *n* = 17), in whom a coherence (*K*^2^) level of >0.5 necessary to indicate a valid phase link between heart rate and blood pressure signals was not met [19]. Therefore, BRS spectral analysis was excluded from further analyses. Cross-correlation and regression between systolic blood pressure and R–R intervals over 10 s sliding windows were used to calculate cross-spectral baroreflex sensitivity (xBRS) [19].

### Hyperinsulinaemic–euglycaemic clamp test

A hyperinsulinaemic–euglycaemic clamp was performed before the intervention and 72 h after the last bout of exercise according to a previously described protocol [33]. Whole-body insulin sensitivity (*M* value) was calculated from the difference between mean glucose infusion rates during the last 30 min of the clamp with glucose space correction [33].

### Cardiorespiratory fitness

Each participant underwent an incremental exhaustive exercise test on an electronically braked cycle ergometer (Ergoline Ergometrix 900, Bitz, Germany) at 60 rev/min to determine $\dot{V}{\mathrm{O}}_{2\max }$ by open-air spiroergometry (Masterscreen CPX, Jaeger/VIASYS, Hoechberg, Germany) as previously described [34].

### Statistical analysis

Data are presented as mean ± SD, median (1st and 3rd quartiles) or percentages. Categorical variables were compared using *χ*^2^ test for cross-sectional and McNemar test for prospective analyses and expressed as percentages of participants. Continuous data were assessed using the parametric *t* test or nonparametric Mann–Whitney *U* test for cross-sectional and Wilcoxon test for prospective data. For multiple linear regression analyses, dependent variables with skewed distribution were log_e_-transformed before analyses. All statistical tests were two-sided and the level of significance was set at *α* = 0.05. All analyses were performed using SPSS version 22.0 software (IBM Corporation, Chicago, IL, USA).

## Results

The demographic and clinical data of the participants with NGT and those with type 2 diabetes are listed in Table 1. At baseline, participants with type 2 diabetes had higher HbA_1c_ and lower *M* value levels compared with the NGT group (*p*<0.05), while age and BMI were comparable between the groups. After 12 weeks of HIIT, HbA_1c_ remained higher and *M* value remained lower in participants with type 2 diabetes compared with the NGT group (*p*<0.05). While HbA_1c_ remained unaltered, *M* value increased from baseline to follow-up in the type 2 diabetes group (*p*<0.05). At baseline, $\dot{V}{\mathrm{O}}_{2\max }$ was higher in the NGT group compared with type 2 diabetes participants (*p*<0.05). An increase in $\dot{V}{\mathrm{O}}_{2\max }$ was observed in both groups after 12 weeks of HIIT (*p*<0.05), while the difference in $\dot{V}{\mathrm{O}}_{2\max }$ between the groups was no longer seen at follow-up. Both systolic and diastolic blood pressure decreased in the type 2 diabetes group from baseline to follow-up (*p*<0.05). In accordance with the study protocol, mean BMI did not change over the intervention period in either group. Participants on antihypertensive medication were treated either with ACE inhibitors or with angiotensin II receptor type 1 (AT1) antagonists. In the type 2 diabetes group, 12 participants were treated with metformin, while five received dipeptidyl peptidase 4 (DPP4) inhibitors and one was treated with glimepiride.

**Table 1:** Demographic and clinical characteristics at baseline and after 12 weeks of HIIT

Variable	NGT (baseline)	NGT (follow-up)	T2D (baseline)	T2D (follow-up)
*n* (% male)	23 (100)	23 (100)	20 (100)	20 (100)
Diabetes duration (years)	–	–	5 (2; 9)	5 (2; 9)
Age (years)	57 (53; 60)	57 (53; 60)	56 (53; 63)	58 (53; 63)
BMI (kg/m^2^)	30.3 (28.4; 32.2)	30.2 (28.7; 32.4)	31.2 (28.6; 32.8)	31.2 (28.9; 33.2)
Smoking (%)	0	0	0	0
SBP (mmHg)	128 (122; 132)	124 (117; 139)	133 (123; 152)	129 (124; 144)^‡^
DBP (mmHg)	78 (73; 84)	77 (72; 82)	82 (74; 85)	74 (68; 83)^‡^
Creatinine (μmol/l)	85.8 (81.3; 91.9)	85.8 (76.9; 99.9)	84.9 (72.5; 91.9)	86.6 (77.8; 93.7)^‡^
Triacylglycerols (mmol/l)	1.50 (1.05; 161)	1.45 (0.95; 175)	1.55 (1.29; 2.40)	1.37 (1.12; 1.90)
Cholesterol (mmol/l)	5.87 (5.25; 6.15)	5.38 (4.71; 5.95)*	5.04 (4.29; 6.23)	5.22 (4.22; 5.90)
HDL (mmol/l)	1.34 (1.24; 1.60)	1.29 (1.19; 1.40)	1.14 (1.01; 1.53)	1.24 (1.06; 1.50)
LDL (mmol/l)	4.09 (3.59; 4.40)	3.41 (3.23; 4.06)*	3.44 (2.59; 4.27)	3.36 (2.59; 4.19)
HbA_1c_ (mmol/mol)	35.5 (33.3; 38.8)	35.0 (33.3; 37.7)	51.9 (46.7; 63.7)*	50.3 (47.5; 55.2)^†^
HbA_1c_ (%)	5.4 (5.2; 5.7)	5.4 (5.2; 5.6)	6.9 (6.4; 8.0)*	6.8 (6.5; 7.2)^†^
*M* value (μmol kg^−1^ min^−1^)	33.3 (26.3; 40.8)	39.5 (28.6; 44.0)	13.4 (8.9; 28.0)*	21.0 (11.7; 33.6)^†,‡^
$\dot{V}{\mathrm{O}}_{2\max }$ (ml kg^−1^ min^−1^)	25.5 (23.9; 28.0)	29.6 (26.9; 32.4)*	23.1 (20.5; 26.0)*	27.0 (25.0; 30.1)^‡^
Albuminuria (%)	13	0	25	25
Confirmed DSPN (%)	0	0	23.5	23.5
Borderline/definite CAN (%)	5.6/0	5.6/0	16.7/11.1	5.6/5.6
Antihypertensive drugs (%)	17.4		30.0	
Glucose-lowering drugs (%)	0		85.0	

Data are median (1st; 3rd quartile) or %

*p<0.05 vs NGT (baseline)

^†^*p*<0.05 vs NGT (follow-up)

^‡^*p*<0.05 vs T2D (baseline)

–, Not applicable; DBP, diastolic blood pressure; SBP, systolic blood pressure; T2D, type 2 diabetes

**Fig. 1:**
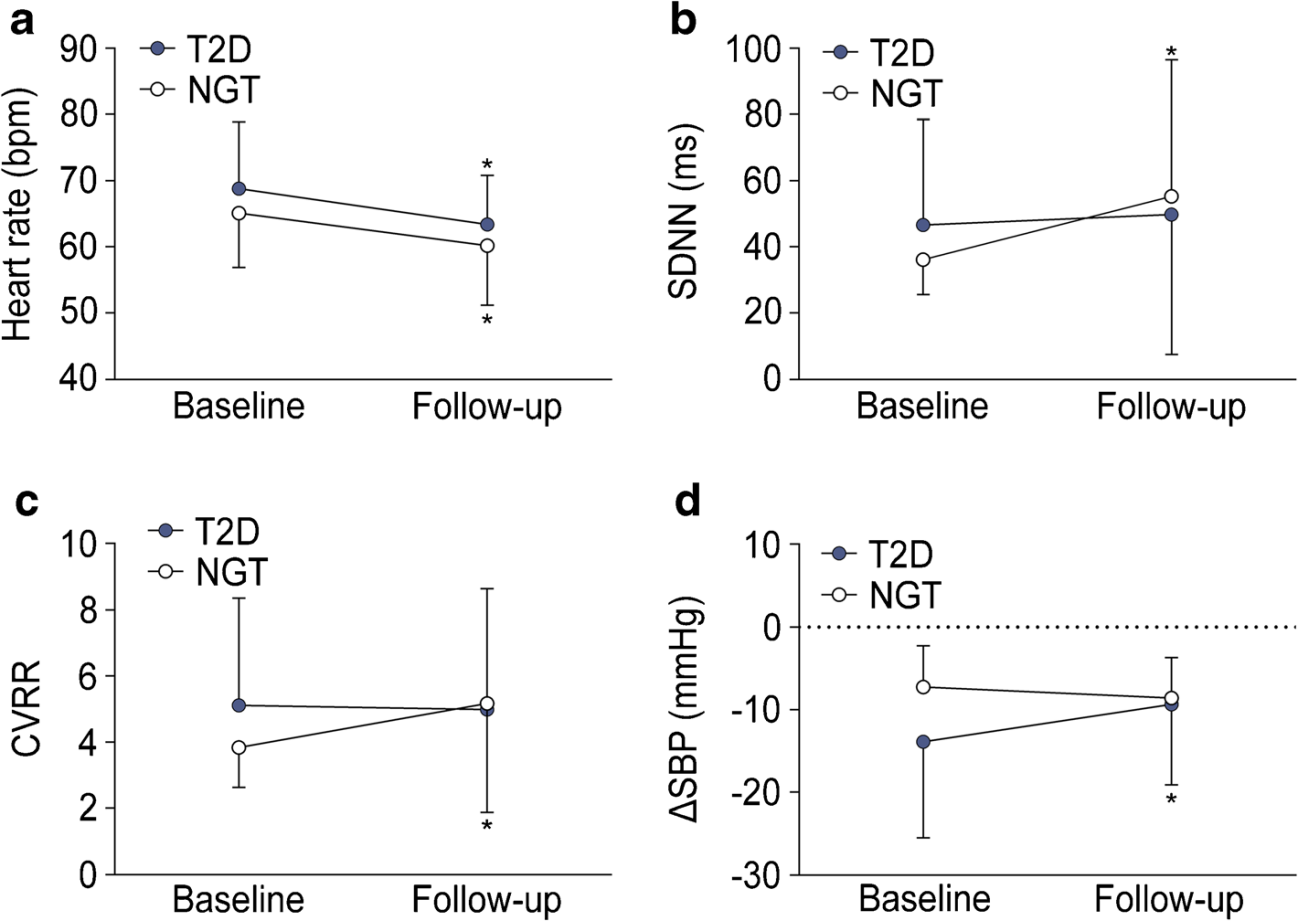
Indices of cardiovascular autonomic function that improved from baseline to 12 weeks in either group or both groups. Blue circles, type 2 diabetes (T2D); white circles, NGT. Heart rate (**a**), SDNN (**b**), CVRR (**c**) and change in systolic blood pressure after standing up from supine position (ΔSBP) (**d**). Data are mean ± SEM; *p≤0.05 vs baseline. bpm, beats per min

**Fig. 2:**
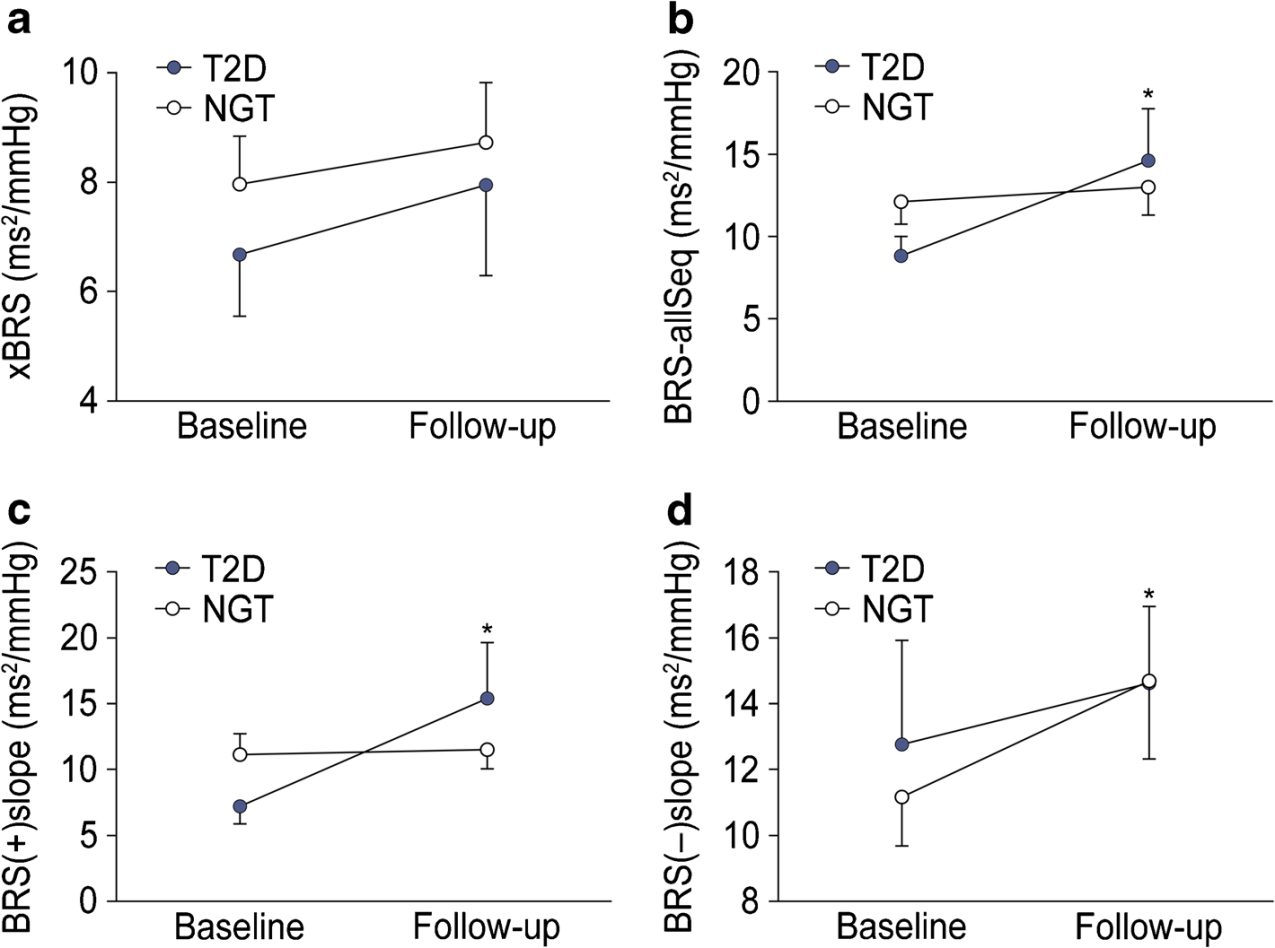
Baroreflex function after 12 weeks of HIIT. Blue circles, type 2 diabetes (T2D); white circles, NGT. xBRS (**a**), BRS-allSeq (**b**), BRS(+)slope (**c**) and BRS(−)slope (**d**). Data are mean ± SEM; *p≤0.05 vs baseline

Somatosensory and autonomic nerve function and IENFD before and after 12 weeks of HIIT are shown in Table 2. After 12 weeks of HIIT, no changes in the somatosensory and autonomic nerve tests listed were observed in either group. Figure 1 shows the four indices of cardiovascular autonomic function which improved from baseline to 12 weeks in either group or both groups. While heart rate improved in both groups ([mean ± SD] baseline/12 weeks: NGT: 65.1 ± 8.2/60.2 ± 9.0 beats per min; type 2 diabetes: 68.8 ± 10.1/63.4 ± 7.8 beats per min), SDNN (36.1 ± 11.8/55.3 ± 41.3 ms) and CVRR (3.84 ± 1.21/5.17 ± 3.28) improved in the NGT group and the change in systolic blood pressure after standing up (ΔSBP) improved (−13.9 ± 11.6/−9.35 ± 9.76 mmHg) in the type 2 diabetes group only (*p*<0.05). The courses of the four BRS indices studied are shown in Fig. 2. Three BRS sequence analysis indices [BRS(+)slope, BRS(−)slope, BRS-allSeq] improved in participants with type 2 diabetes only [BRS-allSeq: 8.82 ± 4.89/14.6 ± 11.7 ms^2^/mmHg; BRS(+)slope: 7.19 ± 5.43/15.4 ± 15.9 ms^2^/mmHg; BRS(−)slope: 12.8 ± 5.4/14.6 ± 8.7 ms^2^/mmHg] (*p*<0.05), whereas xBRS did not significantly improve in either group.

**Table 2:** Peripheral and autonomic nerve function and morphology before and after 12 weeks of HIIT

Variable	NGT (baseline)	NGT (follow-up)	T2D (baseline)	T2D (follow-up)
Median MNCV (m/s)	50.8 ± 4.9	49.3 ± 11.3	50.5 ± 3.0	50.4 ± 3.7
Ulnar MNCV (m/s)	52.6 ± 4.3	52.6 ± 4.7	51.3 ± 5.2	52.2 ± 5.3
Peroneal MNCV (m/s)	43.5 ± 2.9	42.7 ± 0.5	41.5 ± 4.8	40.7 ± 5.3
Median SNCV (m/s)	51.2 ± 5.5	50.3 ± 3.9	48.3 ± 5.6	50.3 ± 5.8
Ulnar SNCV (m/s)	51.3 ± 5.7	48.9 ± 6.5	50.6 ± 6.5	50.3 ± 6.3
Sural SNCV (m/s)	42.1 ± 6.6	41.3 ± 5.6	51.3 ± 5.2	52.2 ± 5.3
Sural SNAP (μV)	8.91 ± 5.73	8.96 ± 7.70	6.83 ± 3.78	7.65 ± 3.46
VPT hand (μm)	0.44 ± 0.21	0.48 ± 0.36	0.74 ± 0.45	0.65 ± 0.31
VPT foot (μm)	3.57 ± 6.23	2.45 ± 2.38	3.20 ± 3.60	3.03 ± 3.93
CDT foot (°C)	26.6 ± 5.7	25.6 ± 8.8	25.3 ± 6.9	26.7 ± 7.0
WDT foot (°C)	39.2 ± 4.1	38.9 ± 4.2	39.0 ± 4.7	38.6 ± 3.9
NSS (points)	0	0	2.65 ± 3.44	0.90 ± 2.27
NDS (points)	0.83 ± 1.07	1.13 ± 1.36	2.00 ± 2.64	1.90 ± 2.00
IENFD (fibres/mm)	7.82 ± 4.72	7.52 ± 3.98	7.00 ± 3.75	7.03 ± 4.23
VLF power (ms^2^)	183 ± 189	2121 ± 7699	319 ± 255	890 ± 2110
LF power (ms^2^)	270 ± 244	2133 ± 7513	591 ± 1271	1798 ± 4858
HF power (ms^2^)	161 ± 160	881 ± 1820	301 ± 539	1065 ± 3004
E/I ratio	1.21 ± 0.99	1.18 ± 0.11	1.19 ± 0.10	1.23 ± 0.17
30:15 ratio	1.21 ± 0.99	1.35 ± 0.15	1.19 ± 0.10	1.31 ± 0.20
Valsalva ratio	1.57 ± 0.32	1.57 ± 0.37	1.45 ± 0.29	1.48 ± 0.30

Data are mean ± SD

CDT, cold detection threshold; E/I ratio, expiration/inspiration ratio; T2D, type 2 diabetes; WDT, warmth detection threshold

In the overall cohort, correlations were observed between the change in two out of three BRS sequence analysis indices and the change in $\dot{V}{\mathrm{O}}_{2\max }$ after 12 weeks of HIIT [BRS(−)slope: *r* = 0.665, *p*=0.003; BRS-allSeq: *r* = 0.528, *p*=0.017]. The associations remained statistically significant after adjustment for age, BMI and HbA_1c_. No associations of the changes in measures of DSPN and CAN after 12 weeks of HIIT with HbA_1c_, *M* value, serum lipids, BMI or the change in systolic blood pressure over 12 weeks were found for the entire cohort or in the individual groups after adjustment for age, BMI and HbA_1c_ (data not shown).

## Discussion

The results of this study demonstrate an improvement in spontaneous BRS and orthostatic blood pressure regulation after 12 weeks of supervised HIIT without weight reduction in overweight or obese male individuals with type 2 diabetes. Moreover, while resting heart rate improved in both groups with and without type 2 diabetes, two HRV measures improved only in the latter group. In contrast, no changes in somatosensory nerve function or IENFD were found, suggesting that cardiovascular autonomic innervation is more susceptible to this short-term intervention. It is also conceivable that 12 weeks of HIIT may not be long enough to induce favourable effects on peripheral nerve function and/or structure.

While several exercise interventions showed that physical activity may improve cardiovascular autonomic regulation in type 2 diabetes [35], studies assessing the effects of HIIT on cardiovascular autonomic nerve function in patients with diabetes are scarce. A systematic review by Batacan et al [23] recently reported that HIIT was more effective in reducing both resting blood pressure and heart rate in studies examining obese (BMI ≥30 kg/m^2^) rather than lean participants but only if the training period lasted for ≥12 weeks. Our results indicating a slight improvement in resting heart rate and blood pressure in overweight or obese men with type 2 diabetes after 12 weeks of HIIT are compatible with this notion. A higher resting heart rate represents an independent risk factor for an increased mortality rate [36] and may indicate sympathovagal imbalance, with resting tachycardia representing a serious complication of advanced CAN [37]. Hence, a reduction in resting heart rate may be a relevant factor in the prevention or treatment of CAN. The reduction in resting heart rate and improved orthostatic blood pressure regulation observed herein in participants with type 2 diabetes may be attributed to an improved baroreflex function [19, 23] mirrored by improvement in BRS indices. No correlations were found between 12 week changes in autonomic function and measures of glycaemic control, insulin sensitivity, cholesterol or BMI. However, correlations between the changes in BRS indices and $\dot{V}{\mathrm{O}}_{2\max }$ over 12 weeks of HIIT were observed in the entire cohort, suggesting a link between improved cardiorespiratory fitness and baroreflex function. This indicates that HIIT exerts favourable effects primarily on baroreflex function linked to improved cardiorespiratory fitness and thus may reduce cardiovascular risk in obese patients with type 2 diabetes independent of defining characteristics of the metabolic syndrome. A previous study reported improved measures of BRS and HRV in response to an oral glucose load after 16 weeks of aerobic ET in obese adults [38]. Slight improvements in cardiorespiratory fitness and BMI were observed in obese participants without but not with diabetes. In a recent small randomised controlled open-label trial [21], unsupervised HIIT over 12 weeks improved glycaemic control, but not BMI or measures of HRV or BRS. Overall, long-term supervised HIIT should be considered to achieve beneficial effects on cardiovascular autonomic regulation in patients with type 2 diabetes.

Since only limited data are available on the effects of HIIT on peripheral nerve function in patients with diabetes, a comparison of published studies with ours is difficult. Hamed and Raoof [39] reported a better effect of HIIT compared with moderate-intensity aerobic training after 15 weeks in reducing neuropathic pain in obese women with type 2 diabetes and polyneuropathy. In the present study, no improvement in neuropathic symptoms was observed. However, the percentages of individuals in the type 2 diabetes group with DSPN or symptomatic DSPN, respectively, may have been too small to observe beneficial effects on neuropathic symptoms. Moreover, no conclusions about effects of HIIT on symptoms in female individuals with DSPN can be drawn from the present data due to the exclusively male cohort studied herein.

No changes in nerve conduction studies or QST were observed following 12 weeks of HIIT. Previous studies addressing the effects of lower intensity ET on peripheral nerve function in diabetes reported contrasting results. Kluding et al [11] did not observe differences in nerve conduction velocity or QST after 10 weeks of aerobic and strengthening ET in participants with DSPN. Proximal and distal IENFD remained unaltered, while more branching was observed in skin biopsies at the proximal site. Gholami et al [40] reported increased sural SNCV after 10 weeks of aerobic ET along with lower fasting glucose and HbA_1c_ levels. In a long-term aerobic ET study by Balducci et al over 4 years [9], a lower percentage of people with diabetes performing prescribed and supervised moderate ET for 4 h/week developed DSPN as compared with the standard diabetes care group, suggesting that long-term ET could indeed modify the natural history of DSPN. Notably, differences between the groups were observed only after 2–4 years, casting doubt on the notion that exercise interventions may improve peripheral nerve function in humans with diabetes within a few months only [2]. In the present study, HbA_1c_ levels and BMI remained unchanged over 12 weeks of HIIT and nerve conduction tests were largely within the normal range at baseline. These factors may have made it more difficult to achieve improvements in somatosensory nerve function. By contrast, cardiovascular autonomic function, particularly BRS, might be more susceptible to HIIT, possibly due to its link to cardiorespiratory fitness [34]. This notion is supported by the association of improvements in BRS measures with increased $\dot{V}{\mathrm{O}}_{2\max }$ after 12 weeks observed herein. Future studies should examine potential effects of HIIT on peripheral nerve function and neuropathic symptoms including pain in larger cohorts focusing on individuals with DSPN.

Intraepidermal nerve fibre regeneration after exercise intervention has been studied previously with a chemical axotomy model using capsaicin patches to denervate the dermis [41]. In a supervised ET study by Singleton and colleagues [41], IENFD regeneration rate was comparable in obese individuals with and without type 2 diabetes and reinnervation rate improved after the intervention. However, a greater degree of reinnervation was observed in those who achieved improvement in multiple metabolic syndrome components and was associated with improved glycaemic control, but not with better BMI or triacylglycerol levels. In accordance with our study, no improvement in IENFD per se was observed after 6 months, suggesting that long-term effects on nerve fibre morphology may require longer periods of increased physical activity.

The strengths of the present study are: first, the comprehensive peripheral nerve assessment including objective large and small fibre function tests, small fibre morphometry and clinical examination; second, the comprehensive battery of cardiovascular autonomic nerve function tests; and, third, the supervised state-of-the-art HIIT intervention programme in two metabolically different groups of individuals. Moreover, effects of weight loss were excluded as participants were instructed to maintain their body weight during the study. Limitations of the study include the moderate sample size of exclusively male individuals and the fact that most participants did not present with impaired peripheral or autonomic nerve function. Hence, changes in nerve function may have been more difficult to detect compared with a more homogeneous group of patients with clinically manifest diabetic neuropathy and/or CAN. Moreover, beneficial effects may have been more pronounced in a combined exercise and dietary intervention to improve body composition and glycaemic control.

In conclusion, after 12 weeks of HIIT, improvements in spontaneous BRS and orthostatic blood pressure regulation rather than somatosensory nerve tests were observed in the absence of weight reduction in overweight or obese men with type 2 diabetes. These results suggest that HIIT may be a useful therapeutic addition to improve cardiovascular autonomic function and cardiovascular risk. It remains to be established whether longer training periods of HIIT may exert positive effects on peripheral nerve function and structure in diabetes. Supervised HIIT programmes may help to achieve beneficial effects of increased physical activity, especially in obese individuals with diabetes.

## Data Availability

Original data are available from the corresponding author on reasonable request.

## Abbreviations

30:15 ratio: Lying to standing maximum/minimum 30:15 ratio
BRS: Baroreflex sensitivity
BRS-allSeq: All-sequences BRS
BRS(+)slope: Positive-sequences BRS
BRS(−)slope: Negative-sequences BRS
CAN: Cardiovascular autonomic neuropathy
CVRR: Coefficient of R–R interval variation
DSPN: Diabetic sensorimotor polyneuropathy
ET: Exercise training
HF: High-frequency
HIIT: High-intensity interval training
HRV: Heart rate variability
IENFD: Intraepidermal nerve fibre density
LF: Low-frequency
MNCV: Motor nerve conduction velocity
NDS: Neuropathy Disability Score
NGT: Normal glucose tolerance
NSS: Neuropathy Symptom Score
QST: Quantitative sensory testing
ΔSBP: Postural change in systolic blood pressure
SDNN: Standard deviation of R–R intervals
SNAP: Sensory nerve action potential
SNCV: Sensory nerve conduction velocity
VPT: Vibration perception threshold
VLF: Very low-frequency
xBRS: Cross-spectral baroreflex sensitivity
